# Characterization of Alginate–Gelatin–Cholesteryl Ester Liquid Crystals Bioinks for Extrusion Bioprinting of Tissue Engineering Scaffolds

**DOI:** 10.3390/polym14051021

**Published:** 2022-03-03

**Authors:** Alyaa Idrees Abdulmaged, Chin Fhong Soon, Balkis A. Talip, Siti Adibah Ahmad Zamhuri, Salama A. Mostafa, Wenbin Zhou

**Affiliations:** 1 Future Food Research Group, Faculty of Applied Sciences and Technology, Universiti Tun Hussein Onn Malaysia, Pagoh Higher Education Hub, Panchor 84600, Johor, Malaysia; alyaaflayh3@gmail.com (A.I.A.); balkis@uthm.edu.my (B.A.T.); 2 Microelectronics and Nanotechnology-Shamsuddin Research Centre, Institute for Integrated Engineering, Universiti Tun Hussein Onn Malaysia, Parit Raja, Batu Pahat 86400, Johor, Malaysia; adibazamhuri@gmail.com; 3 Faculty of Electrical and Electronic Engineering, Universiti Tun Hussein Onn Malaysia, Parit Raja, Batu Pahat 86400, Johor, Malaysia; 4 Faculty of Computer Science and Information Technology, Universiti Tun Hussein Onn Malaysia, Parit Raja, Batu Pahat 86400, Johor, Malaysia; salama@uthm.edu.my; 5 Department of Mechanical Engineering, Imperial College London, London SW7 2AZ, UK

**Keywords:** tissue engineering, 3D extrusion bioprinting, bioink, biomaterials, alginate, gelatin, cholesteryl ester liquid crystals

## Abstract

Tissue engineering (TE) is an innovative approach to tackling many diseases and body parts that need to be replaced by developing artificial tissues and organs. Bioinks play an important role in the success of various TE applications. A bioink refers to a combination of a living cell, biomaterials, and bioactive molecules deposited in a layer-by-layer form to fabricate tissue-like structures. The research on bioink attempts to offer a 3D complex architecture and control cellular behavior that improve cell physical properties and viability. This research proposed a new multi-material bioink based on alginate (A), gelatin (G), and cholesteryl ester liquid crystals (CELC) biomaterials, namely (AGLC) bioinks. The development of AGLC was initiated with the optimization of different concentrations of A and G gels to obtain a printable formulation of AG gels. Subsequently, the influences of different concentrations of CELC with AG gels were investigated by using a microextrusion-based 3D bioprinting system to obtain a printed structure with high shape fidelity and minimum width. The AGLC bioinks were formulated using AG gel with 10% weight/volume (*w*/*v*) of A and 50% *w*/*v* G (AG10:50) and 1%, 5%, 10%, 20%, and 40% of CELC, respectively. The AGLC bioinks yield a high printability and resolution blend. The printed filament has a minimum width of 1.3 mm at a 1 mL/min extrusion rate when the A equals 10% *w*/*v*, G equals 50% *w*/*v*, and CELC equals 40% *v*/*v* (AGLC40). Polymerization of the AGLC bioinks with calcium (Ca^2+^) ions shows well-defined and more stable structures in the post-printing process. The physicochemical and viability properties of the AGLC bioinks were examined by FTIR, DSC, contact angle, FESEM, MTT assay, and cell interaction evaluation methods. The FTIR spectra of the AGLC bioinks exhibit a combination of characteristics vibrations of AG10:50 and CELC. The DSC analysis indicates the high thermal stability of the bioinks. Wettability analysis shows a reduction in the water absorption ability of the AGLC bioinks. FESEM analysis indicates that the surface morphologies of the bioinks exhibit varying microstructures. In vitro cytotoxicity by MTT assay shows the ability of the bioinks to support the biological activity of HeLa cells. The AGLC bioinks show average cell viability of 82.36% compared to the control (90%). Furthermore, cultured cells on the surface of AGLC bioinks showed that bioinks provide favorable interfaces for cell attachment.

## 1. Introduction

Mostly, tissues and organs for transplantation have come from donations but mostly are not suitable in meeting clinical needs. According to the organ procurement and transplantation network (OPTN), there is a wide gap between the number of organs in demand and donated organs for transplantation, and this difference keeps significantly growing year by year [1,2,3]. To meet the enormous need for tissue and organ for transplantation, tissue engineering (TE) has emerged as an alternative and promising solution to develop and fabricate bio-substitutes of tissues and organs that can be used [4,5]. The main principles of TE include the combination of cells, biomaterials, and engineering technologies that are used to engineer tissue substitutes with biological functions that simulate the functions of the natural tissue in the human body [3,6]. TE combines three-dimensional (3D) matrices, also known as scaffolds, cells, bioactive molecules, and growth factors to engineer 3D functional tissue-like constructs. The 3D matrices provide structural and mechanical support for the regeneration process. They have the capability to restore, replace, maintain, or improve the functionality of damaged tissues or failing organs [7,8]. The proper design of the 3D matrix is a key aspect to guide the formation of new functional tissue [9]. The optimal 3D scaffold should finely replicate the physicochemical properties of the native tissue in vitro. Thus, the scaffold induces construct integration in the host tissue while providing proper cues toward cells interactions by directing their behavior and differentiation toward the desired phenotypes [10,11].

3D bioprinting is one of the most applied technologies in the field of tissue engineering, the most prominent one being the biofabrication of 3D tissue and organ models. Advances in 3D bioprinting have made it possible to mimic the complexity and heterogeneity of the native tissues [9,12,13]. Bioinks play an essential role in 3D bioprinting; they provide structural and mechanical supports for improving cells attachment, proliferation, and differentiation of tissue lineages [14,15,16]. To engineer a 3D microenvironment that better mimics the complexity of native tissues, the selection of appropriate materials for the scaffold is essential for ensuring the success of any fabricated construct. A variety of both natural and synthetic polymers have been utilized in the fabrication and development of the bioinks, such as alginate, poly (ethylene glycol) (PEG), gelatin, hyaluronic acid, collagen, chitosan, cellulose, and polyvinyl alcohol (PVA), to produce complex biomimetic structures [17].

A single biomaterial in bioinks cannot meet all mechanical and functional requirements, which are vital to produce biomimetic tissue-like constructs. Multi-materials bioink refers to the combination of more than one type of biomaterial, one or more than one type of cells, and bioactive molecules [9,18,19]. Multi-material (or multi-component) bioinks are currently gaining interest for 3D bioprinting to improve the performance of biomimetic structures. Alginate is a linear hydrophilic polysaccharide naturally derived from brown seaweed. Alginate is an anionic polymer, chemically composed of a combination of (1–4)-linked *β*-D-mannuronic acid (M) and *α*-L-guluronic acid (G) monomers [20,21]. Alginate-based bioinks were widely investigated and used in designing and fabricating 3D cell matrices [22]. This is because the alginate has attractive features for biomedical applications, including excellent biocompatibility, non-immunogenic effect, shear-thinning property, and ease of gelation with divalent cations such as calcium ions (Ca^2+^) [23,24,25]. However, one of the challenges of using alginate-based bioinks is their limited inherent cell adhesion and cellular interaction [18,26,27]. To tackle this limitation, the modification of alginate-based bioinks by the addition of polymers containing cell-binding sides, such as gelatin, has been demonstrated as an effective way to increase cells’ biological activities of printed structures [28,29]. Gelatin is a natural, biocompatible, and biodegradable protein obtained from partial hydrolysis of collagen (the most profuse protein in the extracellular matrix (ECM) of the mammalian). This material exhibits appropriate biological properties that can support cell growth and function, thereby displaying suitable properties for bioprinting applications [28,30,31]. The blending of alginate with gelatin has significantly impacted the viability of different types of cells and printing criteria [32].

In addition to the existing class of synthetic biopolymeric materials, liquid crystal (LC) forms a unique class of synthetic biomaterials. This type of crystal shows intermediate phases between the liquid and solid-state (called liquid-crystalline or mesophases), with diverse chemical compositions and properties [33,34]. LCs have widespread industrial applications of modern technologies and commodity products, such as electronic display devices, biosensors, lubrication, optical devices, and drug delivery systems [35,36]. The LCs have a wide range of characteristics and features, such as rapid reorganization of molecules, viscoelasticity, sensitivity to external stimuli, and flexibility. Many researchers from the TE field have been combining other materials with LCs because of their attractive features [37,38]. Moreover, many molecules in the biological system, such as nucleic acid (DNA), cell membrane, proteins, phospholipids, and cholesterols, show liquid crystalline-like phases [39,40]. For this reason, particular attention has been paid to LC polymers to design novel 3D biomimetic microenvironments for TE applications. Du et al. [41] developed chitosan/cholesterol hydroxypropyl cellulose ester liquid crystal (CS/LC) composite hydrogel and found that LC offers consistent spatial orientations that result in the promotion of initial cell adhesion. The LC further offers active sites to the cells at substrate interfaces, increasing cell attachment and spreading and improving ECM secretion. Wu et al. [42] reported that the cellular behavior of human umbilical cord-derived mesenchymal stem cells (hUC-MSCs) cultured on the PU/LC composite substrate was influenced by the variation of the substrate microstructure. Their study has demonstrated the introduction of different contents of LCs to polyurethane (LC-10/PU, LC-30/PU, and LC-50/PU), which have been intensely used by cells to sense the bioactive patterns and viscoelastic properties of LC domains. The cells, anchored to the LC/PU substrates, were also shown to respond to the topographical and mechanical cues, which subsequently modulated the cellular behavior of the hUC-MSCs, such as adhesion, proliferation, and differentiation.

Recently, the combination of alginate and gelatin for 3D bioprinting has been widely attempted because of chemical similarity to the extracellular matrix (ECM) found in native tissues. Di Giuseppe et al. [30] studied alginate/gelatin hydrogels’ mechanical behavior for application in extrusion-based 3D bioprinting. The study investigated the influence of varying the individual constituent concentrations of alginate–gelatin (AG) blends on printability, print accuracy, compressive behavior, and cell proliferation. The results showed that hydrogel prepared with 7% alginate–8% gelatin yielded higher printability with a minimum width of the printed strands of 0.32 mm, and printing accuracy exceeded 90% more than the other formulations of AG hydrogels prepared with lower concentrations. Mechanical properties of the tested hydrogels were increased by increasing the concentrations of both Alg and Gel. The cell survival rate of mesenchymal stem cells (MSCs) encapsulated within AG was found to be 90%, which indicated high cell viability. Othman et al. [43] constructed a multi-layered hexagonal 3D structure with human adenocarcinoma cancer cell lines (HeLa) spheroids in the AG hydrogel. Othman et al. [43] tested different alginate and gelatin solutions viscosities to check printability, mechanical properties, viscoelasticity, and microtissue spheroids viability. The results showed that ALG10-Gel50 bioink (10% *w*/*v* alginate and 50% *w*/*v* gelatin) enables high fidelity printing of the multi-layered 3D construct. The 3D printed structure showed mechanical properties close to the in vivo system, hydrophilicity, and amino groups. 3D microtissue of HeLa spheroids showed high cell viability at 95%. Deoxyribonucleic acid, lipid, and amino acids related to collagen were also characterized. In the previous work of Abdulmaged et al. [32], the printability and 3D printing quality of AG hydrogel with LC have been investigated. The printability results show that the produced bio compounds have good printability properties and the potential of successful bioinks.

Du et al. [41] developed chitosan/cholesterol hydroxypropyl cellulose ester CS/LC composite hydrogel. They found that LC offers consistent spatial orientations that result in the promotion of initial 3T3 fibroblasts adhesion. The LC further offers active sites to the 3T3 fibroblasts cells at substrate interfaces, increasing cell attachment and spreading and improving ECM secretion. Nasajpour et al. [44] explored CLC/PCL nanofiber as a scaffold for C2C12 myoblasts. The scaffold provided the cultured C2C12 myoblasts with a favorable microenvironment for the cell to adhere, migrate, and differentiate. The number of adherent cells increased with increasing CLC bulk concentration (50% *w*/*v*) with high confluent cellular layers’ formation instead of the scaffolds with lower CLC concentrations. The introduction of CLC in different concentrations created a bio-simulating interface that responds to cellular mechanical cues. The PCL polymer provides the scaffold with an elastic bulk matrix. The homogeneously dispersed CLC establishes a viscoelastic fluid-like interface that imitates the viscoelastic properties of the native ECM. Multi-material bioinks are formulated with a combination of different biomaterials such as alginate with gelatin [30,43], alginate, gelatin, fibrinogen hydrogel [45], and alginate–chitosan [46]. They provide simple ECM analogs for the cell to recapitulate partial bio-functionality of native human tissues. The bioactivity of natural human tissue depends upon several additional native ECM features such as the lipid that remain challenging to incorporate into bioink formulation [10]. The lipid is an essential element for the cell membrane and stabilizing bilayer lipid membrane [47]. These characteristics provide biologically active functional groups for cells to anchor and induce cellular-level mechano-biological physiological functions found in tissues. However, incorporating lipid into bioink formulation is one of the significant challenges of TE [10].

This paper proposed a new multi-materials bioink of alginate–gelatin–cholesteryl ester liquid crystal (AGLC) and its respective polymers. The reason behind selecting these biomaterials is that the combination of alginate and gelatin has been widely used due to its chemical similarity to the extracellular matrix (ECM) of native tissues. The CELC was selected due to its biocompatibility and shear-thinning properties. The literature has included several studies such as Nasajpour et al. [44] and Soon [48] that state the ability of the CELC to provide lipid moieties. Hence, combinations of these biomaterials may produce bioinks that can simulate a certain degree of natural tissues and serve as a platform for TE applications. The design model of the AGLC bioinks consists of alginate (A), gelatin (G), and CELC. Alginate was selected as the main material for the bioink because of its good biocompatibility and mild crosslinking with divalent ions. Gelatin was also used to enhance cell attachment, spreading, and proliferation while CELC endowed lipid moieties. Subsequently, the proposed multi-material AGLC bioinks were characterized in terms of the printability, physicochemical, and cell viability properties to imitate the complexity of natural tissues. A microextrusion-based 3D bioprinting system was used to test the printability of the AGLC bioinks experimentally. The physicochemical properties of the bioinks were characterized by Fourier-transform infrared spectroscopy (FTIR), differential scanning calorimetry (DSC), field emission scanning electron microscopy (FE-SEM), contact angle measurement, and MTT assay. The cell viability of the bioinks was evaluated using MTT Assay and 2D culture.

## 2. Materials and Methods

**Figure 1:**
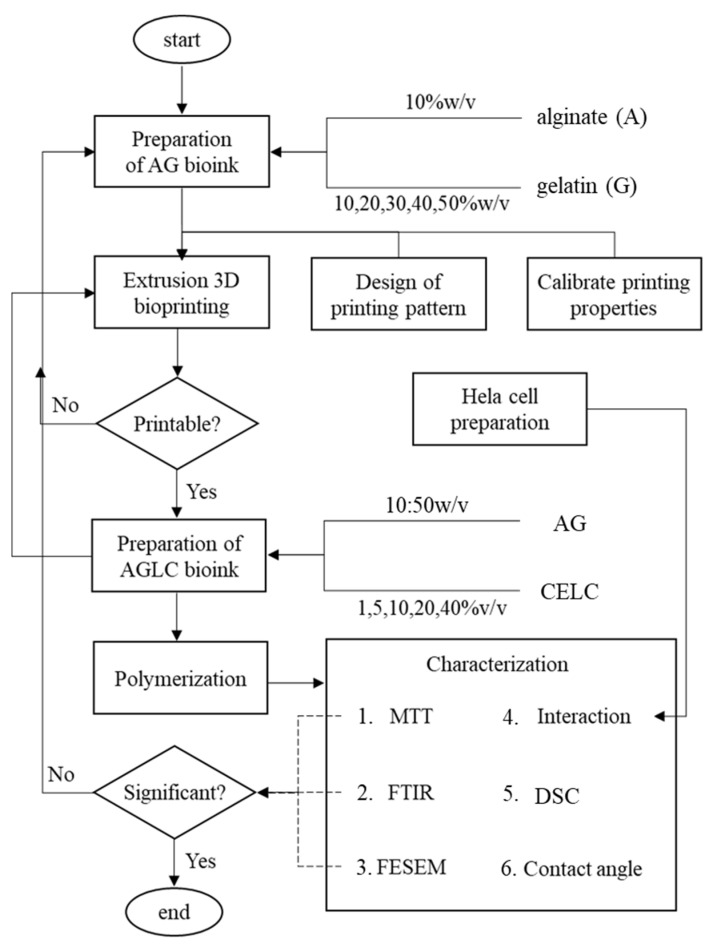
The flow chart of the research activities. The top part of the figure includes the printability of AG hydrogel. The middle part includes the printability of the AGLC bioinks. The bottom part of the figure shows the characterization of the AGLC bioinks.

This work attempts to develop and optimize multi-material alginate–gelatin–CELC bioink for 3D cell culture. The work started with the optimization of alginate–gelatin (AG) gels to produce a printable pattern with high resolution and shape fidelity using a microextrusion-based 3D printer. Subsequently, the optimized AG gel was used to synthesize the AGLC bioink by adding CELC in different concentrations based on the microextrusion 3D printer. This step was to study the effect of CELC on the printability of AGLC bioink. Then, the physicochemical properties of AGLC bioink were characterized using different methods such as FTIR, DSC, FESEM, contact angle measurement, and MTT assay. The characterization was conducted to understand the effect of CELC on the chemical and thermal properties, surface morphology, wettability, and cell survival rate of the bioink. Figure 1 shows the main research components and activities of this work.

The research scope includes the preparation of AG and AGLC bioinks in different concentrations and preparation of 3D extrusion bioprinting. It requires designing printing patterns, calibrating printing properties, and characterization of the bioinks. The characterization encompasses FTIR, DSC, contact angle measurement, FESEM, MTT assay, and cell interaction. All results were presented as mean ± standard deviation (SD). The obtained data from FTIR and DSC were interpreted using OriginLab Pro software. A statistical comparison of MTT assay data was analyzed by *t*-test. *p* ˂ 0.05 was considered to be statistically significant, n = 3.

### 2.1. Preparation of Materials

#### 2.1.1. Preparation of Alginate–Gelatin (AG) Hydrogels

The gel solution of A at the concentration of 10% *w*/*v* and G solutions at different concentrations of 10–50% *w*/*v* were prepared. In a clean and sterilized beaker, 1 g of sodium alginate powder (Sigma-Aldrich, St. Louis, MO, USA) was dissolved into 10 mL of deionized water. Then, 1 g of G powder (Sigma-Aldrich, USA) was dissolved in 10 mL of deionized water under constant stirring at 60 °C. Then, the solution of 10% *w*/*v* A and the solution of 10% G were blended and centrifuged at 1200 rpm for 1 min to remove air bubbles. To prepare AG10:20, 2 g of G powder was dissolved in 10 mL of DI water, and 3, 4, and 5 g were dissolved separately in 10 mL of DI water to prepare 30%, 40%, and 50% *w*/*v* of G solutions, respectively. Table 1 shows the prepared AG hydrogels with different concentrations of G solutions. The concentration in weight/volume percentage (*w*/*v*)% was calculated using Equation (1). The AG solutions were denoted as AG10:10, AG10:20, AG10:30, AG10:40, and AG10:50 according to their corresponding concentrations.

(1) $$(w/v)\%=\frac{weigh\;of\;solute\;in\;\left(gram\right)}{volume\;of\;solution\;in\;\left(ml\right)}\times 100$$

#### 2.1.2. Preparation of Cholesteryl Ester Liquid Crystals Biomaterial

The cholesteryl ester liquid crystals (CELC) biomaterial sample used in this work was formulated using 37.5% of cholesteryl oleyl carbonate, 37.5% of cholesteryl perlargonate, and 25.0% of cholesteryl chloride (Sigma, Aldrich, St. Louis, MO, USA). It was prepared in a glass vial; the solid mixtures of the three LCs were heated on a Cimarec™ digital stirring hotplate (Thermo Scientific, SP131320-33Q, Waltham, MA, USA) to their liquid phase (isotropic phase) at the temperature range between 115–120 °C according to Soon [48]. It comprises the physical solids of (a) cholesteryl oleyl carbonate, cholesteryl percarbonate, and cholesteryl chloride; (b) the liquid phase of the CELC mixture; and the (c) liquid crystalline phase of the CELC mixture.

#### 2.1.3. Preparation of Alginate–Gelatin–CELC

To prepare AG–CELC Bioinks (AGLC) bioinks, the CELC gel (at liquid-crystalline phase) contained within a glass vial was melted again at 80 °C on a hotplate to its isotropic phase (liquid phase). Different CELC sample (isotropic phase) volumes were added to previously prepared AG10:50 hydrogel using a micropipette, as shown in Table 2.

Table 2 shows the AGLC bioinks prepared with different concentrations of CELC. The concentration in volume/volume percentage (*v*/*v*)% of AGLC bioinks was calculated by Equation (2) below.

(2) $$\left(\frac{V}{v}\right)\%=\frac{volume\;of\;lC\;in\;\left(ml\right)}{volume\;of\;AG\;in\;\left(ml\right)}\times 100$$

Subsequently, the blends of AGLC bioinks were stirred manually to obtain homogeneous solutions denoted as AGLC01, AGLC05, AGLC10, AGLC20, and AGLC40. Then, all AGLC bioinks synthesized at different concentrations of CELC were centrifuged for 1 min at 1200 rpm to remove trapped air in the bioinks solutions.

#### 2.1.4. Preparation of Crosslinking Solution Calcium Chloride

Calcium chloride solution was used at the concentration of 1% as a crosslinker in the polymerization of AGLC bioinks to stabilize the printed structures. Calcium chloride solution (CaCl_2_) was prepared under sterile conditions by dissolving 0.6 g of calcium chloride anhydrous, granular, ≤7.0 mm, ≥93.0% (Sigma-Aldrich, USA) into 60 mL of distilled water to obtain 1% Calcium chloride. The solution was shaken well for 1 min to ensure that CaCl_2_ granules were well-solubilized into distilled water. Equation (3) below was used to calculate the concentration in the percentage of CaCl_2_ solution.

(3) $$\left(w/v\right)\%=\frac{weigh\;of\;solute\;in\;\left(gram\right)}{volume\;of\;solution\;in\;\left(ml\right)}\times 100$$

#### 2.1.5. Cell Preparation and Subculture

HeLa cells were used throughout this study to test the biocompatibility of AGLC bioink owing to the simplicity of handling and culturing these cells. The HeLa cell line has been widely used in the development of cancer models and anti-cancer therapeutics (Othman et al., 2020; Liu et al., 2018). Moreover, this cell line is also used to test the biocompatibility of different types of scaffolds such as agarose chitosan coated silver nanoparticles scaffold for tissue engineering application and cellulose–alginate composite scaffold (Kumer et al., 2018 and Ku et al., 2010). HeLa cells used in this research were purchased from (ATCC, Manassas, VA, USA). The cells were maintained in a 25 cm^2^ tissue-treated culture flask containing Gibco^R^ Dulbecco’s Modified Eagle’s Medium with high glucose (Sigma Aldrich, Gillingham, UK). The culture medium used was supplemented with 10 mL of L-glutamine, fetal bovine serum (50 mL, Sigma Aldrich, UK), penicillin-streptomycin (5 mL, Sigma Aldrich, Gillingham, UK), and amphotericin (1 mL, Sigma Aldrich, Gillingham, UK).

Upon reaching 80% of confluency, 1 mL of trypsin was added to the cells contained in the culture flask. Then, the cells were incubated in a CO_2_ incubator for 5 min. After trypsinization, the flask was visually inspected in a Nikon Eclipse TS100 phase-contrast microscope installed with a Go-5 charged-couple device (CCD) camera to ensure that most of the cells were detached from the surface of the plastic. A total of 5 mL of supplemented culture media was added to arrest the trypsinization process. The cells were removed from the culture flask and deposited into a 15 mL tube before being centrifuged at 1000 rpm for 5 min. After centrifugation, the old media was discarded, and the cell pellet was re-suspended in 5 mL of supplemented culture media. Then, the cells were ready for the following experiment.

### 2.2. 3D Printing System

**Figure 2:**
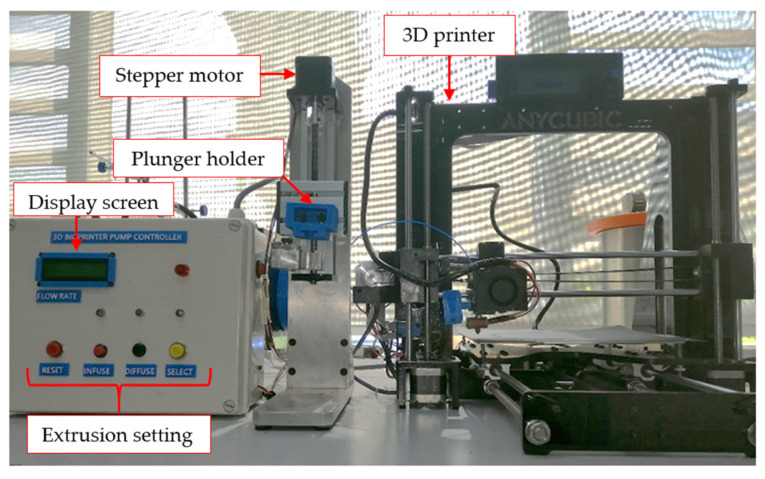
The 3D microextrusion-based bioprinting system used in the bioinks printability test.

The used 3D microextrusion-based bioprinting system for bioink fabrication in this study was developed in our lab. As shown in Figure 2, the 3D bioprinting instrumentation system consists of an extruder pump controller, a customized extruder machine, and a commercial 3D printer (Anycubic, Tsim Sha Tsui, Kowloon, Hong Kong, China). The fabrication process and printability test of AG hydrogels (AG10:10, AG10:20, AG10:30, AG10:40, and AG10:50) and AGLC bioinks (AGLC01, AGLC05, AGLC10, AGLC20, AGLC40) were performed as follows. First, AG hydrogel was loaded into a disposable syringe. The syringe was fixed on a costume-made syringe holder on the extruder machine (as shown in Figure 2) and connected with a silicon tube fitted with a printing nozzle with a 1 mm inner diameter. The printing nozzle was fixed on a plastic holder on the 3D printer. For the printability test, a simple 2D shape (square shape) with one layer was designed using the open-source Inkscape software version 0.92.3. This software can be used to design any kind of shape and convert it into a G-code file. The designed structure is then saved and converted into a G-code file, which is acceptable for the 3D printer. Appendix A shows the 3D bioprinter main components and the bioprinting process of AG hydrogels and AGLC bioinks. The G-code directs the printing head to create the square shape using a series of G, X, Y, Z commands. A segment of the G-code is used to print a square with a width of 3.5 cm × 3.5 cm × 3.5 cm.

## 3. Experiments

The development and optimization of AGLC multi-material bioinks for 3D cell culture were conducted on a microextrusion 3D bioprinting system. Experiments were performed to investigate the influence of gelatin and CELC concentration on the printability of the bioinks.

### 3.1. Printability Experiments

#### 3.1.1. Printability Experiments of Alginate–Gelatin Hydrogels

The purpose of this experiment is to optimize the printability of AG hydrogel. Printability refers to the ability to form a 3D structure with good fidelity, structural integrity, and cell viability. It represents a major component in this study by blending 10% of alginate solution with different contents of gelatin solutions as a supportive biomaterial to improve the mechanical properties of the alginate hydrogel. In this experiment, alginate concentration was fixed at 10% *w*/*v*. The gelatin concentration was varied from 10–50% *w*/*v*. The printability of AG hydrogels was conducted as described in Section 3.2.

#### 3.1.2. Printability Experiments of AGLC Bioinks

The purpose of these experiments is to study the influences of the CELC with different concentrations (1, 5, 10, 20, and 40) on the printability of AG10:50 hydrogels (filaments width and resolution). These experiments include five formulations of AGLC bioinks. The first printability experiment was conducted with the formulation at 1% of CELC (AGLC01), the second experiment included the formulation of 5% of CELC (AGLC05), the third and fourth experiments included the formulation of 10% and 20% of CELC (AGLC10 and AGLC20), respectively, and the fifth experiment was conducted with the formulations of 40 of CELC (AGLC40).

### 3.2. Characterization Experiments

The characterization included five types of experiments. The characterization of the AGLC bioink included the investigation of the physicochemical properties and cytotoxicity of the bioink.

#### 3.2.1. Polymerization

The printed scaffolds of AGLC bioink were still in the gel phase and required to be solidified. The entire printed structures of AGLC bioinks were immersed in 1% CaCl_2_ solution for 15 min at room temperature (25 °C). This experiment was conducted to investigate the ability of the Ca^2+^ ions to crosslink the alginate chains in the presence of CELC at different concentrations to form more stable structures of AGLC bioinks post-printing.

#### 3.2.2. Fourier Transform Infrared Spectroscopy

**Figure A2:**
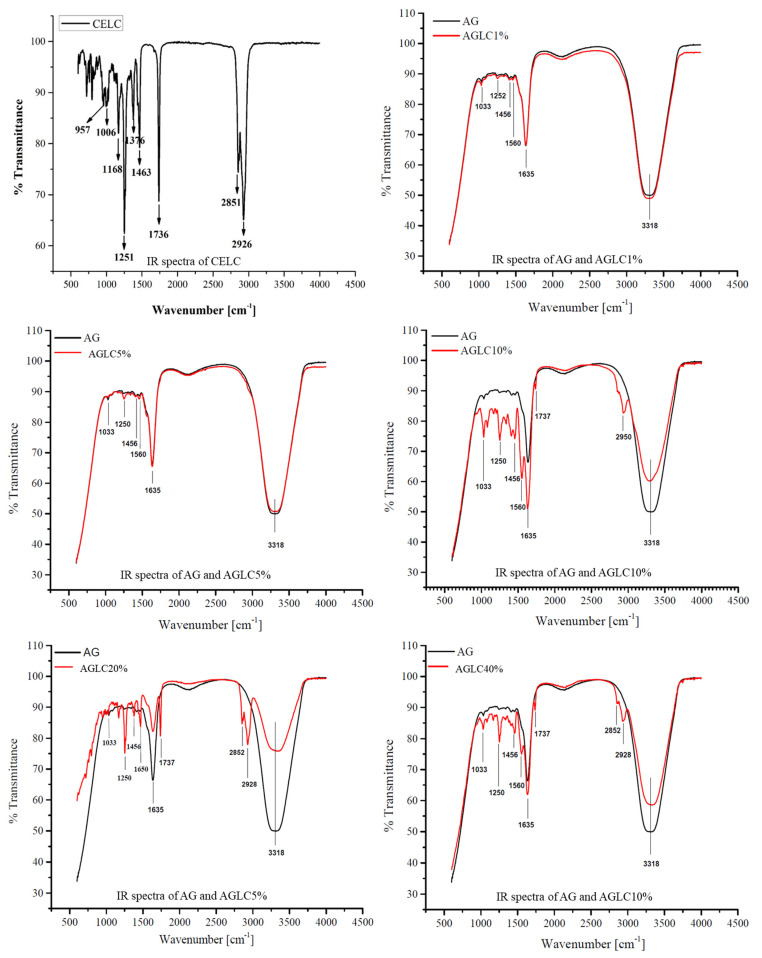
IR spectra of the bioinks.

In this test, the chemical properties of polymerized AG and AGLC bioinks were conducted using FTIR spectroscopy (PerkinElmer Spectrum100, Waltham, MA, USA) fitted with an attenuated total reflection assembly (ATR). All samples for the FTIR test were prepared as mentioned in Section 3.2.1 and Section 3.2.2 and polymerized with 1% of CaCl solution for 15 min. Before the scan, the samples were cut into small pieces and placed onto the sample stage under the universal attenuated total reflectance (UATR). Subsequently, the pressure arm was positioned over the samples. The IR spectra of AG, CELC, and AGLC bioinks were recorded in the ranges of 4000–600 cm^−1^ at a spectral resolution of 4 cm^−1^ and a scan rate of 32 scans per second (see Appendix B, Figure A2).

#### 3.2.3. Differential Scanning Calorimetric

Differential scanning calorimetric (DSC, Q20, USA) analysis and its derivatives were used to study the thermal stability and thermal properties of AGLC bioinks at different contents of CELC. Polymerized AGLC and AG bioink samples were prepared as reported in Section 2.1.3. Approximately 8–12 mg of each sample was weighed and placed in an aluminum pan, tightly covered by an aluminum lid, and heated at a rate of 10 °C/min. An empty aluminum pan with its lid was prepared as a reference for the DSC test. All measurements were carried out under nitrogen flow at a temperature range from 0–250 °C.

#### 3.2.4. Contact Angle Measurement

In order to analyze the surface wettability of AG and AGLC bioinks, contact angle measurement was performed using the sessile drop technique water contact angle VCA-optima instrument (AST Inc., Billerica, MA, USA). Samples of AG10:50 and AGLC bioinks were prepared as described in Section 2.1.3 and polymerized with 1% of CaCl_2_ solution for 15 min. Thereafter, all the samples were fixed on a glass slide, and 10 µL of deionized water dropped onto the surface of the samples, and an image of the droplet was recorded.

#### 3.2.5. Field Emission Scanning Electron Microscopy

Field emission scanning electron microscopy (FE-SEM) (Jeol JSM-7600F, Akishima, Tokyo, Japan) was used to evaluate the change in surface morphology of AGLC bioinks. Prior to the examination, a sample of AG10:50 and AGLC bioinks (AGLC01, AGLC05, AGLC10, AGLC20, and AGLC40) were air-dried at room temperature (25 °C), and the surface of the samples was coated with a thin layer of gold using a JFC-1600 auto-fine coater (JEOL. Tokyo, Japan) sputtering coater.

#### 3.2.6. In Vitro Cytotoxicity of AGLC Bioinks

In vitro cytotoxicity of AGLC bioink and its respective polymers (AGLC01, AGLC05, AGLC10, AGLC120, and AGLC40) was investigated using the 3-(4,5-dimethylthiazol-2-yl)-2,5-diphenyltetrazolium bromide (MTT) assay. MTT, a water-soluble yellow tetrazolium, reduces water-insoluble formazan crystals in living cells by cellular dehydrogenase. The amount of formazan developed is directly proportional to the number of living cells.

HeLa cells were seeded in a 96-well culture plate at a density of 10^4^ cells/well and were incubated at 37 °C for 24 h. After incubation, the old media was removed, and 100 μL of fresh media was added with AGLC bioinks samples cut in the size of 2 mm × 2 mm. The treated cell samples with AGLC were incubated for 24 h. Samples of the AGLC bioinks were removed from the 96-well culture plate. Subsequently, the 96-well plate was rinsed with 100 μL of Hank’s Balanced Salt Solution (HBSS) (Sigma Aldrich, USA). Finally, 10 μL of MTT assay was added to the wells, and cells were further incubated for 2 h. After 2 h, the MTT solution was collected, and 100 μL of dimethyl sulfoxide (DMSO) was added. The absorbance was determined at 540 nm using a microplate reader (Multiskan GO, Germany). For the purpose of the reference, cells were seeded into a fresh culture medium (control) with the same seeding conditions. Cell viability (%) was calculated as in Equation (4).

(4) $$\mathrm{Cell}\;\mathrm{viability}\;\%=\left(\frac{\left[abs\right]\;sample-\left[abs\right]\;blank}{\left[abs\right]\;control-\left[abs\right]\;blank}\right)\times 100$$

where [*abs*] *sample*, [*abs*] *control*, and [*abs*] *blank* represent the absorbance values for the cells treated with materials, cells treated with culture medium, and absorbance of the blank solution, respectively.

#### 3.2.7. Cell Interaction

This experiment aims to observe the interactions between HeLa cells and the surface of the AGLC bioinks. In this experiment, AGLC bioink samples were prepared, as mentioned in Section 2.1.3. Subsequently, AGLC samples were polymerized for 15 min into 1% of CaCl_2_ solution and placed into a small petri dish. Then, 1 mL of suspended HeLa cell in DMEM medium was added to the surface of the samples cut in the size of 1 mm × 1 mm. After treatment with the cells, 2.5 mL of DMEM culture media was deposited onto the samples and incubated for 24 h at a condition of 37 °C and humidified environment (5% CO_2_). Images of the AGLC samples with and without cell treatment were snapped under a Nikon Eclipse TS-100 inverted phase-contrast microscope.

## 4. Results

### 4.1. Printability Results

#### 4.1.1. Printability of AG Hydrogels

**Figure 3:**
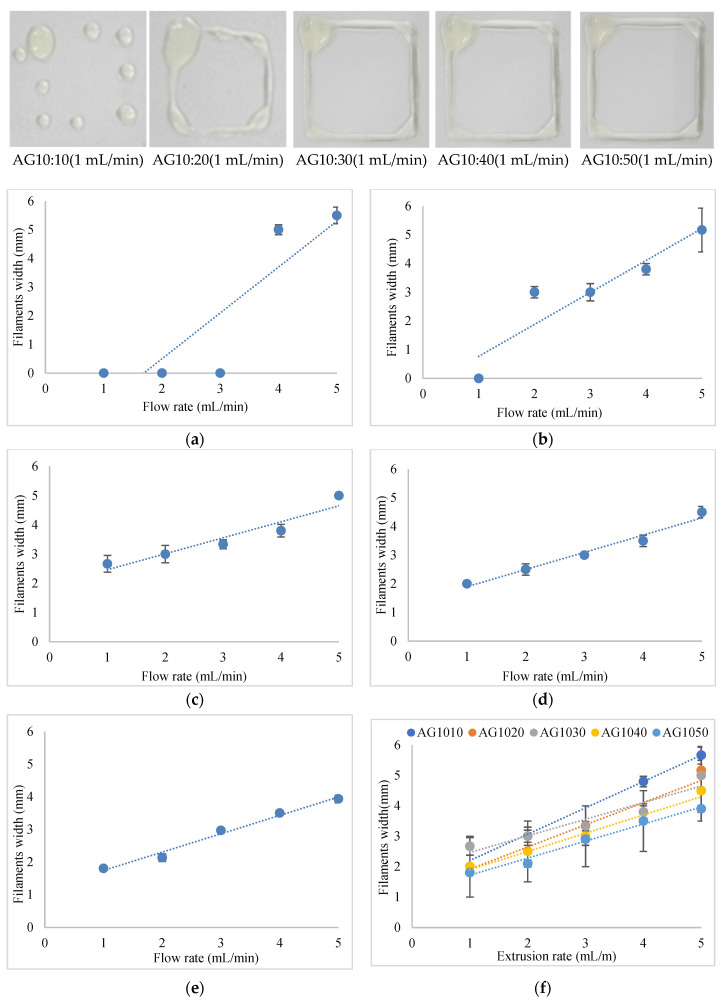
The results of the printed pattern of AG10:10–50 gel blends at different flow rates (1–5 mL/min) using an extrusion-based 3D bioprinter. (**a**) AG10:10; (**b**) AG10:20; (**c**) AG10:30; (**d**) AG10:40; (**e**) AG10:50; (**f**) AG10:(10–50).

In the first phase of this work, we study and show the effects of adding G concentrations to A to construct variations of AG hydrogels. Five experiments were conducted to investigate the printability of the AG hydrogel. The first experiment was initiated with a blend of 10% *w*/*v* alginate and 10% *w*/*v* gelatin (AG10:10). The second experiment was performed with the hydrogel of 10% *w*/*v* alginate and 20% *w*/*v* gelatin (AG10:20). The third experiment was performed with a blend of 10% *w*/*v* alginate and 30% *w*/*v* gelatin (AG10:30). The fourth experiment was performed with a blend of 10% *w*/*v* alginate and 40% *w*/*v* gelatin (AG10:40). The fifth experiment was conducted with a blend of 10% *w*/*v* alginate and 50% *w*/*v* gelatin (AG10:50). The sample results of these experiments are shown in Figure 3.

It can be seen from Figure 3 that the gel prepared with 10% *w*/*v* of gelatin (AG10:10) was unprintable at the flow rate of 1–3 mL/min and exhibited an incomplete pattern of the printed structures. Subsequently, at higher flow rates of 4 and 5 mL/min, the deposited blend of AG10:10 spread unacceptably and showed a high extend of the printed filaments, resulting in an undefined shape of the printed design. These results suggested that the hydrogel prepared with 10% *w*/*v* gelatin has low viscosity and cannot hold the designed pattern’s printed shape despite the applied low or high flow rates.

Based on Figure 3, it can be observed that, at a 1 mL/min flow rate, the AG10:20 gel was printable but produced in an incomplete pattern of the printed structure. Increasing the extrusion rate of the AG10:20 gel from 2–5 mL/min resulted in an increase in the width of the printed structures filaments, and this is due to the low viscosity of the gel prepared with 20% *w*/*v* of gelatin, which caused the width of the filaments to be increased. Figure 3 also shows that the width of the filament of the AG10:30 hydrogel increased linearly from 2.5 to 3.8 mm with an increase in the extrusion rate from 1–4 mL/min, and the printed shape showed better printing accuracy than those in AG10:10 and AG10:20. The width of the filament increased to 5.0 mm when the flow rate of 5 mL/min was applied. Increasing the gelatin concentration to 30% *w*/*v* has likely increased the viscosity of the AG10:30 hydrogel and improved printability. However, the viscosity of the printed gel (AG10:30) is still insufficient to achieve high printing accuracy and thinner filaments (<2 mm).

Preferably, the width of the printed filament is close to the width of the orifice of the nozzle at 1 mm. The filament of the printed structure of AG10:40 hydrogel demonstrated a linear relationship between the flow rate and the filament width compared with the previously examined gels. The minimum width of the printed filaments achieved with this formulation was 2.0 mm when the flow rate was set at 1 mL/min. The results of this experiment suggested that increasing the concentration of gelatin to 40% resulted in an increase in the viscosity of the AG10:40 hydrogel and reduced the extent of filaments spreading. The width of the filament of the printed structure of the AG10:50 (i.e., 10% *w*/*v* alginate and 50% *w*/*v* gelatin) gel decreased, and a well-defined lining of the printed structure was observed as the concentration of gelatin increased to 50% *w*/*v*. It is also observed that the standard deviation of printed filaments decreased with the AG10:50 gel. The thinnest filament width line achieved in this experiment was 1.8 mm when the flow rate was set at 1 mL/min. Clearly, with the higher viscosity of the hydrogel blend, the printed structures are better defined and lower width size. This has increased the thixotropy property of the 3D printing of alginate/gelatin.

These results imply that the bioinks printing fidelity is associated with the viscosity of the composite bioinks. Printability results of AG hydrogels containing low concentrations of gelatin 10–30% *w*/*v* exhibited liquid-like extrusion and poor features to hold the printed shape due to insufficient viscosity. Thus, they failed to produce well-printed structures, making them unfeasible to manufacture a 3D construct with mechanical strength. There is a relationship of filament width to the extrusion rate of different concentrations of AG gels. The gelatin concentration above 40% *w*/*v* plays an important role in increasing the viscosity and printability of the gels. On the other hand, increasing the concentration of gelatin to 40% and 50% (AG10:40 & AG10:50) increased the viscosity and decreased the flow of the hydrogels, which offered high printing consistency and better control of printed filaments width and maintained the post-printing structural stability. In particular, the AG10:50 hydrogel printability exhibited high printing resolution (thin filaments) and well-defined shape post extrusion to support the printed pattern and possessed the appropriate viscosity to maintain the 3D shape than AG10:40 hydrogel.

A similar trend on the printability of AG hydrogels at low proportions (7% *w*/*v* Alginate—8% *w*/*v* gelatin) was previously demonstrated by Di Giuseppe et al., 2018, by printing a strand with a single layer of the material. A printability (strand thickness and accuracy) test showed a decrease in strand width with increasing concentration of gelatin solutions, while the mechanical properties (compressive modulus) of the hydrogels were found to increase with increasing the concentration of gelatin. Their results suggested that the increase of the viscosity was due to the increased concentration of the gelatin since gelatin has the most solid-like characteristic (storage modulus G’ dominates), and hence, gelatin provided the initial viscosity and mechanical stability required for the ink to be printed smoothly onto the platform and hold its shape post-printing. Our results have shown that AG10:50 at 1 mL/min extrusion rate displayed excellent shear-thinning property, extrudability, and shape fidelity after deposition. Therefore, AG10:50 was selected to develop AG and AGLC bioink. Further experiments with CELC were conducted to investigate the effects of CELC at different concentrations on the printability of AGLC bioinks.

#### 4.1.2. Printability of AGLC Bioinks

**Figure 4:**
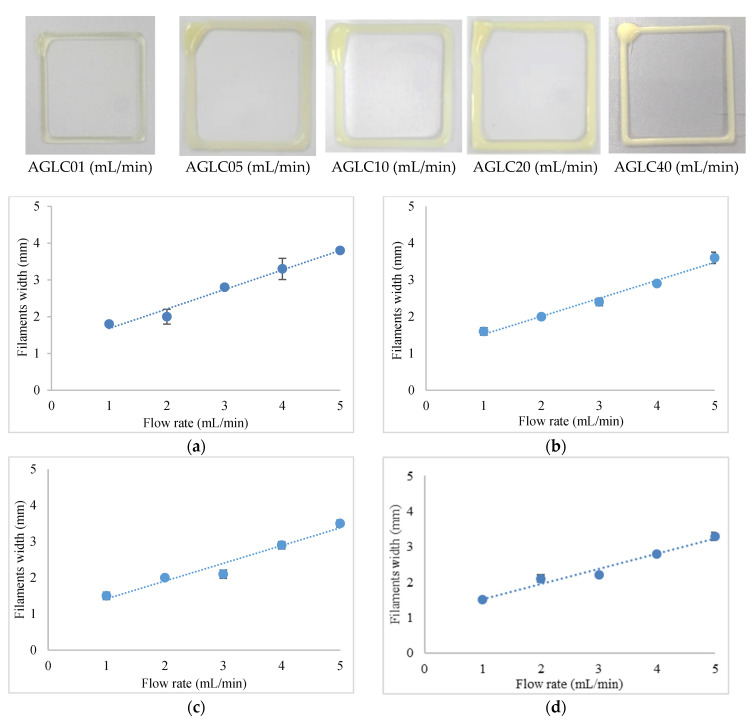

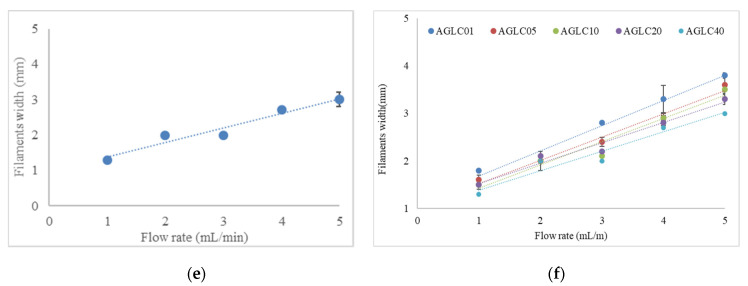
The relationship between the filament width to the flow rate of AGLC bioinks. (**a**) AGLC01; (**b**) AGLC05; (**c**) AGLC10; (**d**) AGLC20; (**e**) AGLC40; (**f**) AGLC bioinks.

We show the effects of CELC concentration on the printability of AG hydrogel by conducting five experiments. These experiments include five formulations of bioinks with different concentrations of CELC. The five-printability experiment were conducted with a blend of 10% *w*/*v* alginate–50% *w*/*v* gelatin and 1%, 5%, 10%, 20%, and 40% *v*/*v* CELC. Figure 4 shows the results of the printed pattern of AGLC01, AGLC05, AGLC10, AGLC20, and AGLC40 bioinks at different flow rates (1–5 mL/min). Figure 4 shows the relationship between the filament width to the flow rate of AGLC bioinks at different flow rates (1–5 mL/min).

### 4.2. Characterization Results

This section depicts the characterization process and the obtained results of the AGLC bioinks. The characterization of AGLC bioinks includes polymerization, chemical properties, thermal properties, wettability, surface morphology, and cytotoxicity.

#### 4.2.1. Polymerization

**Figure 5:**
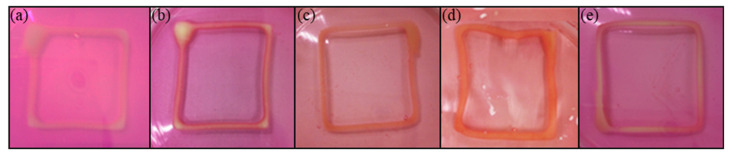
The polymerized structures of (**a**) AGLC01, (**b**) AGLC05, (**c**) AGLC10, (**d**) AGLC20, and (**e**) AGLC40.

Based on Figure 5, the entire printed structures of AGLC bioinks prepared at different concentrations of the CELC showed that the modulated bioinks did not liquefy or disassociate after they had been immersed in 1% of CaCl_2_ solution for 15 min. This result indicated that the Ca^2+^ ions could bind to guluronate (G-blocks) found in the chemical structure of naturally derived alginate. The structure of the G-block in alginate chains offers a high degree of coordination with the divalent cations [22]. The G-block of one polymer binds with the guluronate blocks of the neighboring polymer chains in which it is called the egg-box model of crosslinking, resulting in a three-dimensional network structure [23,49]. It indicated that the crosslinking process successfully inhibited CELC chain mobility and trapped it during the stabilization process of the network [36]. The crosslinking process between Ca^2+^ ions and the guluronate unites of alginate chains was not influenced by the presence of CELC, especially at the high concentrations of the AGLC bioink, i.e., 20% and 40%. These may be due to the high solubility of CaCl_2_ in an aqueous solution. The diffusion of Ca^2+^ ions into the entanglements of alginate chains continued with the presence of CELC at 20% and 40% *v*/*v* concentrations (AGLC20 and AGLC40 bioinks). Figure 5 shows the AGLC bioinks printed frames immersed in DMEM culture media.

#### 4.2.2. Chemical Properties

**Figure 6:**
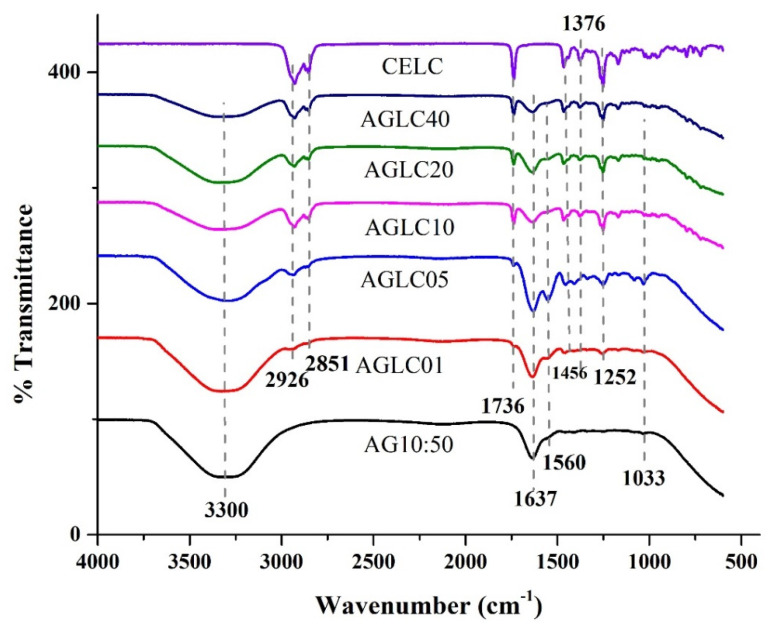
FTIR spectra of AGLC bioinks.

FTIR spectra of polymerized AG10:50 (10% *w*/*v* of alginate and 50% *w*/*v* of gelatin) hydrogel, pure CELC, and AGLC bioinks are shown in Figure 6. The FTIR spectrum of AG10:50 hydrogel in Figure 6 shows a characteristic peak at 3300 cm^−1^ corresponding to O-H stretching vibration. The absorption bands at 1637 and 1409 cm^−1^ were assigned to the carbonyl (C=O) group of asymmetric stretching vibration of carboxylate (COO) and symmetric stretching vibration of COO, respectively [50]. The characteristic peak observed at 1560 cm^−1^ was assigned to the formation of polyelectrolyte (CONH_2_) complex through the electrostatic interaction between the negative charge of COO group in alginate and positive charge of an amine group (NH^+^) in gelatin.

The two strong absorption peaks at 2926 and 2851 cm^−1^ are corresponding to asymmetric C–H and symmetric C–H bands, respectively, present in alkyl groups such as methyl and methylene. The characteristic absorption peak at 1736 cm^−1^ has corresponded to the carbonyl (C=O) group of lipid ester. The absorption bands observed at 1463 and 1376 cm^−1^ were related to the C=C asymmetric stretching vibration of the aromatic ring and stretching vibration of C–O, respectively. The absorption bands below 1200 cm^−1^ were assigned to the fingerprint of LC [51]. According to this figure, the IR spectra of the AGLC bioinks prepared with 1%, 5%, 10%, 20%, and 4% of CELC show both IR characteristic features of the AG hydrogel and the CELC.

As shown in Figure 6, all bioinks exhibit peaks at 3300, 2926, 1637, 2851, 1736, 1637, 1560, 1456, 1252, and 1033 cm^−1^. The wide absorption band at 3300 cm^−1^ corresponds to the stretching vibration of the O–H group. This peak is broadened in the IR spectra of AGLC prepared with 10%, 20%, and 40% of CELC as a result of water repulsion, caused by the hydrophobic nature of the CELC in the bioinks. The two adjacent small peaks observed at 2926 and 2851 cm^−1^ are assigned to symmetric and asymmetric C–H stretching from the CELC. The characteristic absorption bond at 1736 cm^−1^ was assigned to the C=O stretching vibration of lipid ester found in CELC. The strong absorption band at 1635 cm^−1^ was assigned to the asymmetric COO^−^ stretching bond of the carboxylate group from AG hydrogel. The peak at 1560 cm^−1^ corresponds to the formation of the polyelectrolyte complex (CONH_2_) resulting from the electrostatic interaction between the negative charge of the alginate COO^−^ salt group and the positive charge of the gelatin amino group. The absorption bands observed at 1456, 1376, and 1252 cm^−1^ correspond to the symmetric bending vibration of the methyl group of amino acid residues of AG hydrogel and C=C asymmetric stretching vibration of the aromatic ring and stretching vibration of C–O of CELC, respectively. The two peaks at 1243 and 1033 cm^−1^ were related to the NH bending vibration of amide III and C-O group of guluronic units of AG hydrogel, respectively [52,53]. From the peaks of the AGLC bioinks spectra, it was found that the mixtures of AG hydrogel and CELC did not cause any peak shifts of the individual components from both AG hydrogel and CELC.

The importance of CELC arises from the presence of the lipid ester functional group, which has been successfully introduced to the AGLC bioinks as shown in Figure 6 (peak at 1737 cm^−1^), which may provide a better biological activity for the AGLC bioinks. CELC is characterized by cholesteryl moieties that have a universal affinity for cell membranes, which may closely simulate the in vivo conditions [45,54]. The cholesteryl moieties could play an important role in cell membrane formation and stabilization of the bilayer lipid membrane [47]. Hence, the modification of the AG10:50 bioink with different concentrations of CELC may provide a favorable microenvironment that supports cellular activities such as adhesion, migration, proliferation, and differentiation.

#### 4.2.3. Thermal Properties

**Figure 7:**
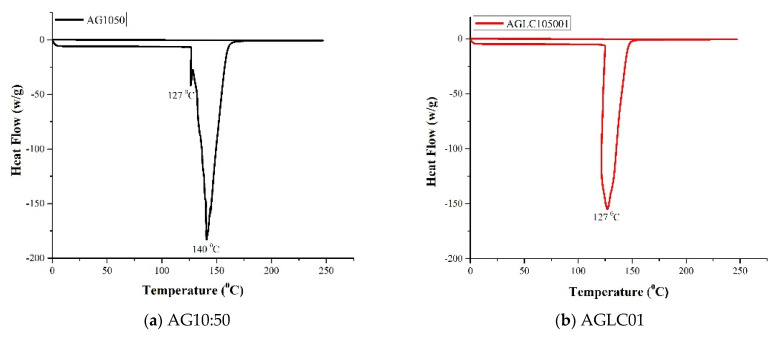

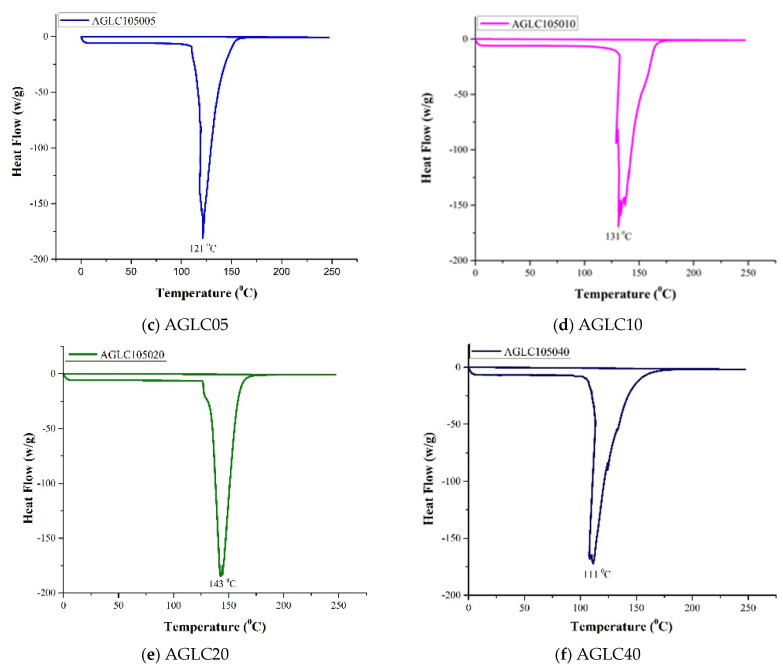
The DSC curves of (**a**) AG10:50, (**b**) AGLC01, (**c**) AGLC05, (**d**) AGLC10, (**e**) AGLC20, and (**f**) AGLC40.

The differential scanning calorimeter (DSC) curves of all AGLC bioinks samples with various concentrations of CELC (AGLC01, AGLC05, AGLC10, AGLC20, and AGLC40) are shown in Figure 7. Figure 7a shows the thermal properties of polymerized AG10:50 (10% *w*/*v* of alginate and 50% *w*/*v* of gelatin) obtained by DSC.

Thermal analysis of the AG10:50 shows a strong endothermic peak and a small shoulder peak observed at 140 and 127 °C, respectively. The shoulder peak at 127 °C is assigned to the evaporation of absorbed water from the hydrogel, and the strong peak at 140 °C could be due to the thermal degradation of alginate and gelatin polymer backbone [53,54,55]. The DSC curves of AGLC01 and AGLC05 bioinks show melting temperatures decreased to 126.9 and 121.2 °C, respectively. It could be attributed to the reduction of water molecules that resulted from the hydrophobic nature of CELC [56,57]. On the other hand, the AGLC10 and AGLC20 bioink samples showed an uptrend in the melting temperature (*T*_m_). The *T*_m_ of AGLC10 and AGLC20 were found to be higher than the samples prepared with 1% and 5% of CELC. This is attributed to more required energy for water molecules’ evaporation of the AGLC10 and AGLC20 bioinks. Meanwhile, increasing the concentration of CELC to 40% in AGLC40 showed a downtrend in melting temperature. The *T*_m_ of the AGLC40 shifted to a low temperature at 111 °C. As the concentration of CELC was increased to 40%, the water content in the bioink was decreased, which in turn reduced the heat required for the decomposition of the AGLC40 bioink. This is in line with FTIR results and the water contact angle. Table 3 summarizes the results of thermal analysis of the neat AG1050 and AGLC bioinks samples.

As shown in Table 3, an increase in the CELC content in the AGLC bioinks correspondingly increased the enthalpies of the system. This indicates that more energy is required for water evaporation from the bioinks. Furthermore, CELC is thermosensitive material, and when temperature increases are applied, the material gradually turns into an isotropic phase. Based on the thermal analysis profile of AGLC bioinks, the AGLC bioinks can be deemed chemically stable at 37 °C, in which there was no sign of endothermic and exothermic activity. This would enable their application in cell culture at incubation temperature (37 °C).

#### 4.2.4. Contact Angle Properties

**Figure 8:**
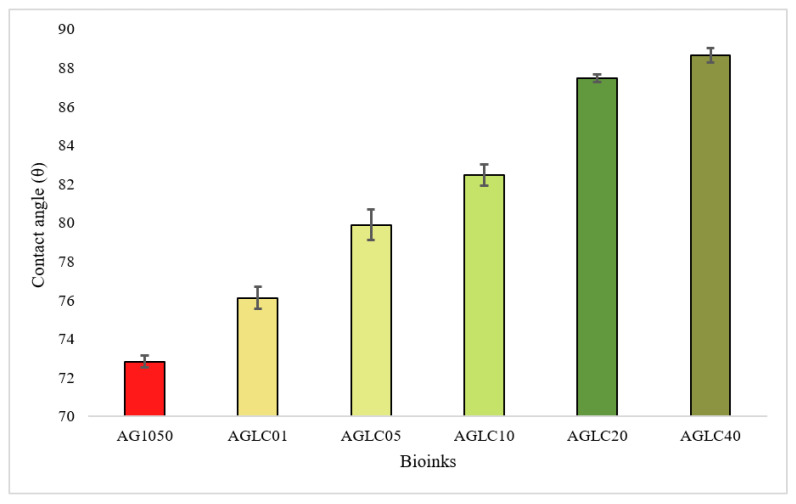
Comparison of contact angle values of AGLC bioinks with varying CELC concentrations.

Contact angle measurement determined the surface wettability (hydrophilicity) of AG10:50 bioinks and AGLC hydrogel. This parameter can affect the protein adsorption and cellular ability to attach to the surface of the scaffolds [58,59]. Figure 8 shows the wettability measurements of all AGLC bioinks compared to the AG10:50 hydrogel.

**Figure 9:**
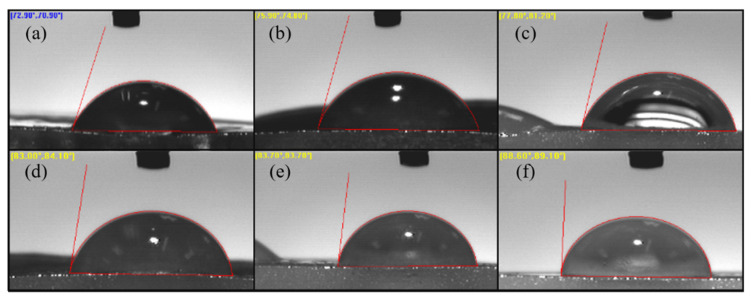
The contact angles of different concentrations of (**a**) AG10:50, (**b**) AGLC01, (**c**) AGLC05, (**d**) AGLC10, (**e**) AGLC20, and (**f**) AGLC40.

According to Figure 8 and Figure 9, an increase in contact angle values of all AGLC bioinks was observed as the concentration of CELC increased (compared with pure AG1050 hydrogel). The values of contact angle measurement show that the AGLC01 bioink is the most hydrophilic sample with a mean value of 76.13° while the bioink prepared with 40% of CELC (AGLC40) has the highest value of contact angle 88.6°. These results show that the addition in CELC highly influenced the hydrophilicity of the modulated bioinks. The introduction of high concentrations of CELC resulted in the formation of more compact structures characterized by low hydrophilicity and interfacial tension of the AGLC bioinks.

Generally, the CELC compound consists of flexible side-chains chemically bonded to a stiff backbone. The stiff backbones tended to orientate, while the soft side chains tended to arrange their order randomly. For different AGLC bioinks, the side chains (esters) of CELC were shorter and consequently less entangled with the soft parts of AG hydrogel. It seems that, through the crosslinking process of AGLC bioinks, the stiff backbone was arranged orderly while the soft esters kept moving, such that the hydrophobic aliphatic side-chains distributed on the surface of the bioinks that made the surface of AGLC bioinks gradually exhibit more hydrophobic feature with increasing in CELC content. Figure 9 shows the contact angles of different concentrations of the AGLC bioinks. An angle lower than 90° reflects the hydrophilic surface and an angle higher than 90° reflects a hydrophobic surface [59,60]. Studies have shown that excessive hydrophilic surface may inhabit protein adsorption and drive out protein molecules, and a high hydrophobic surface may induce strongly irreversible protein adsorption [60,61]. Therefore, this bioink shows moderately hydrophobic properties, which may consider favorable for cell attachment.

#### 4.2.5. Surface Morphology Analysis

**Figure 10:**
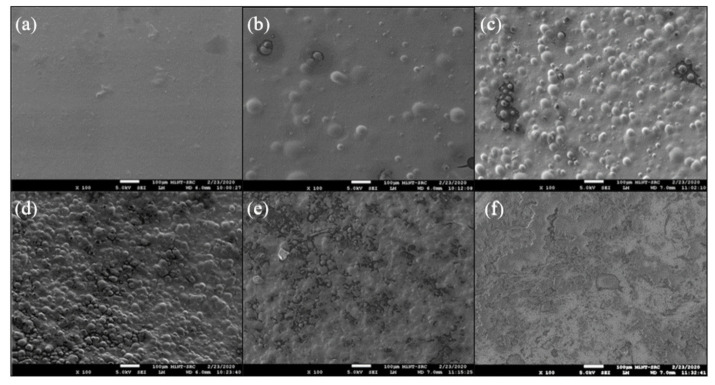
The surface morphologies of (**a**) AG10:50, (**b**) AGLC01, (**c**) AGLC05, (**d**) AGLC10, (**e**) AGLC20, and (**f**) AGLC40.

Figure 10 shows the surface morphology of the pure AG10:50 (10% *w*/*v* of alginate and 50% *w*/*v* of gelatin (a) hydrogel and AGLC bioinks (b–f). It could be seen from the FE-SEM images that the uniform and smooth surface morphology of AG10:50 (Figure 10a) bioinks remarkably altered after the addition of CELC. The roughness of the AGLC bioinks increased as the concentrations of the CELC increased.

The surface morphologies of AGLC bioinks prepared at different proportions of CELC exhibit uniform fine-granulated surfaces, as shown in Figure 10b–f. According to Figure 10b–d, the surface morphologies of AGLC bioinks that prepared at 1%, 5%, and 10% of CELC (i.e., AGLC01, AGLC05, and AGLC10), the CELC formed a scattered, small size island-like droplets dispersed individually throughout the bioinks surfaces. With the increase of CELC content, more rich-CELC domains were generated and gradually gathered and connected, as shown in Figure 10e,f. The FE-SEM images indicated that the surface texture and the micro-morphologies of AGLC bioinks significantly varied after adding CELC to AGLC bioinks. This variation on the surface morphologies of AGLC bioinks could induce different effects on the behavior of different cell types as cells can sense and respond to changes in their surrounding microenvironment, as previously reported in previous studies [41,48,56].

#### 4.2.6. In Vitro Cytotoxicity Properties

**Figure 11:**
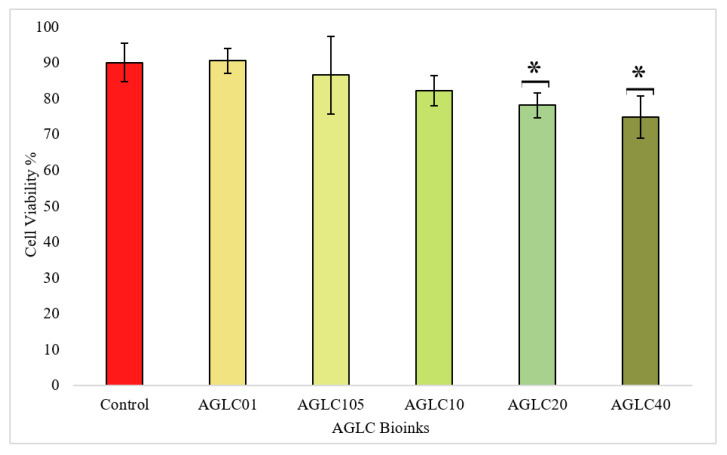
Cell viability % of HeLa cells after incubation with AGLC bioinks for 24 h compared with the control (n = 3, * *p* < 0.05).

The viability of HeLa cells cultured on AGLC bioinks (AG10:50, AGLC01, AGLC05, AGLC10, AGLC20, and AGLC40) with various CELC ratios were evaluated over 24 h of incubation by MTT assay, as shown in Figure 11. Accordingly, the in vitro investigation demonstrated that there are no significant differences between cellular activity and viability of HeLa cells exposed to the AGL01, AGLC05, and AGLC10 bioinks and the control sample. Particularly, the AGLC01 bioink samples showed better cell viability of 90.47% (±3.44) compared to the control (90%). AGLC05 bioink samples achieved cell viability of 86.46% (±10.87) compared with the control. Meanwhile, AGLC20 and AGLC40 exhibited less cell viability of 78.09% (±3.50) and 74.72% (±5.87), respectively. The high concentration of LC of 40% and above has a negative impact on cell viability. Thus, the recommended concentration is 1–20%.

**Figure 12:**
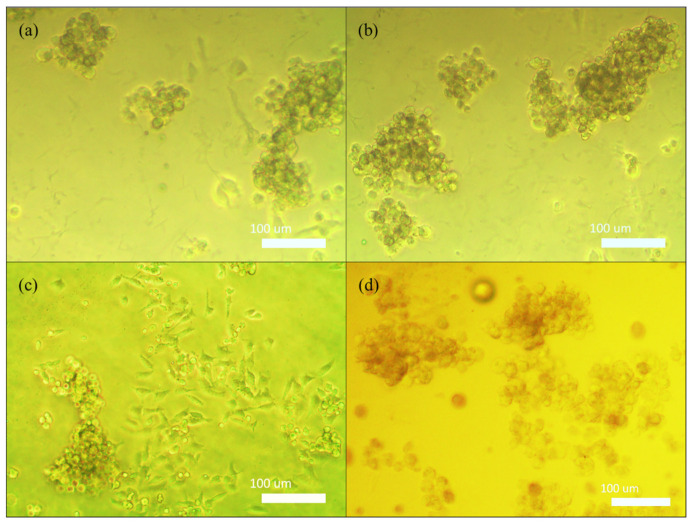
Phase-contrast microscopic images of HeLa cells cultured on the surface of (**a**) AGLC01, (**b**) AGLC05, (**c**) AGLC10, and (**d**) AGLC20 and after 24 h of incubation. Image scale is 100 micrometer (um).

Cell viability results indicated that the AGLC bioinks showed cytocompatibility with HeLa cells, and cells were metabolically active in response to different concentrations of AGLC bioinks. The supportive biocompatibility of AGLC01 is mainly ascribed to the presence of the CELC that may provide vital biological molecules to cells at the bioink interface [47,54]. Although the CELC is well-known for its biocompatibility, cell viability decreased slightly with an increase in the CELC content. Therefore, very high concentrations of the CELC (+50%) may not be useful in constructing bioinks, as they might lead to reducing cell survival rates. The reason might be that the more hydrophobic feature of AGLC bioinks prepared with 20% and 40% *v*/*v* of CELC slows down the diffusion of nutrients and exchanges the catabolites in and out of the bioinks, leading to unhealthy conditions and reduced cell viability [56]. Figure 12 shows the phase-contrast microscopic images of HeLa cells cultured on the surface of the bioinks.

The results of this test infer that the AGLC bioinks with embedded micro-domains of the CELC provided the surface of the substrate with favorable interfaces for cell attachment. Hence, the CELC supports the adhesion of the cells on the surface of the AGLC bioinks. The intrinsic features of the CELC incorporated with the soft matter nature of the AG10:50 hydrogel endowed the AGLC bioinks with biomimetic features suitable for cell adhesion [56,59].

## 5. Discussion

In this research, the fabrication and characterization of the multi-material bioink of AG and AGLC based biomaterials using an in-house microextrusion-based 3D bioprinter system have been successfully achieved. The concentrations of 10–50% *w*/*v* of gelatin solution were investigated with 10% *w*/*v* of alginate solution. The microextrusion-based 3D bioprinting operates at printing speed 30 mm/s^−1^ and extrusion rates 1–5 mL/min to attain the optimum printability results. AG10:50 produced the designed patterns with high resolution and shape fidelity structure among the concentrations above. It achieves the minimum printed width of 1.8 mm at the extrusion rate of 1 mL/min.

Similarly, a microextrusion-based 3D bioprinting system at the same printing parameters was used to test the printability of AGLC bioinks. The bioinks were denoted as AGLC01, AGLC05, AGLC10, AGLC20, and AGLC40%. The addition of CELC biomaterial improves the shear-thinning properties of the AGLC. Hence, the printability of bioinks has been enhanced compared to AG10:50. The printed structure’s high resolution and shape fidelity was achieved when the concentration of CELC was 40%. The minimum width of the printed filaments reaches 1.3 mm at the extrusion rate of 1 mL/min. The outcome has proven that the addition of CELC has a significant effect on the printability of the bioinks.

This objective was carried out by immersing the printed structures immediately into 1% calcium chloride solution for 15 min. The results obtained showed that the printed structures were effectively crosslinked with Ca^+2^ ions during the polymerization process. The crosslinked structures formed through post-fabrication treatment seemed to be more stable than the ones formed without exposure to Ca^2+^ ions and had well-defined shapes. This is because, with the exposure to calcium solution, the guluronic residues in the alginate polymer chain formed crosslink bonds with calcium ions, strengthening the bioinks.

The FTIR results showed successful integration of CELC functional groups into AGLC bioinks, which is important for application in tissue engineering, as the CELC contains an active site for cellular adhesion. FE-SEM images exhibited that the micromorphology of AGLC bioinks noticeably varied with the addition of CELC. These variations in surface micromorphology could cause intense cell responses that might influence cellular behavior, such as adhesion, migrations, and proliferation. DSC analysis has shown that the thermal behavior of the AGLC bioinks is dependent on the bioinks composition. Generally, all the bioinks showed high thermal stability, and they are stable at cell incubation temperatures (37 °C). With the increase of CELC from 0% to 40%, wettability (hydrophilicity) of the AGLC bioinks increased about 26.13% compared to the pure AG bioink (AG10:50) as a result of the hydrophobic nature of CELC. However, all the AGLC bioinks showed moderate hydrophilic characteristics as the values of contact angle were less than 90°. The cell cytotoxicity study is conducted by MTT assay. Statistical analysis on HeLa cell proliferation found that the prepared bioinks with a lower concentration of the CELC were biocompatible and can support the adhesion of the HeLa cell line, as demonstrated by the MTT assay. Furthermore, the cell interaction results inferred that the embedded micro-domains of the CELC provided the surface of the AGLC substrate with more favorable interfaces for cell attachment.

In a nutshell, in the printability-related tests, we have observed that the increase of CELC improved the printability of the AGLC. However, when CELC concentration increases to above 50% *v*/*v*, the bioink becomes difficult to extrude using the 3D bioprinter. On the other hand, in the viability-related test of MTT assay, we have observed that increasing the concentration of CELC by more than 40% *v*/*v* affects cell survivals, as shown in Figure 11. We claim that the AGLC bioink with a range between 5–20% of CELC can achieve more significant results, and our claim needs to be experimentally investigated.

The above experiments showed that the development of a multi-material bioink based on alginate, gelatin, and CELC via extrusion-based 3D bioprinting was achieved. Firstly, the AG bioinks were investigated, and the best printability was achieved when the 10% *w*/*v* alginate was mixed with 50% *w*/*v* gelatin to formulate AG10:50 bioink. This bioink displayed good extrudability and shape-fidelity after deposition. Secondly, the AGLC bioinks were investigated based on AG10:50 bioink and different concentrations of CELC. The results show that the printability of AGLC bioinks was improved as the concentration of the CELC increased. As the concentration of CELC increased, the width of the deposited filament decreased such that the diameter of the printed filament obtained was close to the nozzle diameter used in this work. The best printability was achieved by the AGLC40 bioink when the CELC concentration was 40% *v*/*v*%.

The physicochemical properties of AGLC bioinks were influenced by the CELC. The obtained results showed that wettability decreased as the concentration of CELC increased. The thermal analysis showed that AGLC bioinks are chemically stable at normal incubation temperature (37 °C). The roughness of the AGLC bioinks increased as the concentrations of the CELC increased.

The cell viability test is performed by MTT assay and cell interaction. The MTT assay results showed that the CELC concentration also influenced cell growth. The AGLC05 and AGLC10 bioinks show the highest metabolic activity. In the cell interaction experiment, HeLa cells were cultured on the surface of the AGLC bioinks. This experiment shows that the AGLC bioinks support the adhesion of the cells on the surface of the bioinks.

In general, this work suggests that the AGLC10 and AGLC20 bioinks are the most promising bioinks since they exhibited appropriate printability and cell viability. These two bioinks have the highest potential to create a suitable environment for cell adhesion out of the tested bioinks of this research. As a result, this work suggests a further investigation of the 5–20% *v*/*v* CELC concentrations with 10% *w*/*v* of alginate and 50% *w*/*v* of gelatin that might lead to more significant results. This work focused on developing AGLC multi-material bioinks for extrusion-based 3D bioprinting and studying the influence of the concentrations of CELC on printability and cell viability. However, this work has several limitations that mainly result from the constraint of the scope of the research. The following discusses the main limitations of the work: The proposed structure design and properties are convenient for testing the AGLC bioinks of our work using the extrusion-based 3D bioprinter. Several printing properties can be investigated, such as studying the effect of different printing speeds and shapes (hexagonal, tubular, and grid) on the printability (filament width and resolution) of the AGLC bioinks and investigating the ability to print the AGLC bioinks in different layers and structures. The second point is to investigate the interaction and viability of 3D encapsulation of HeLa cells encapsulated in the printed structure of AGLC for different periods of time. This work only considers testing the HeLa cell as a testing material for the viability of the bioink due to its availability and ease of cultivation. Other types of cells, such as oral carcinoma cells, might be considered to evaluate the bioinks further.

## 6. Conclusions

This research achieves the fabrication and characterization of alginate–gelatin–cholesteryl ester liquid crystals (AGLC) multi-material bioinks. The AGLC bioinks have been applied to a microextrusion-based 3D bioprinting system for TE applications. The AGLC bioinks with different concentrations of CELC were characterized and compared with alginate–gelatin (AG) bioinks. The physicochemical properties of AGLC bioinks were tuned by the addition of a proper amount of the CELC. The AGLC bioinks were effectively crosslinked with Ca^2+^ ions during polymerization. The FTIR results indicated that the CELC functional groups were successfully introduced to AGLC bioinks, which is important for application in tissue engineering, as the CELC contains micro-domains for cells adhesion. FE-SEM images showed that the addition of CELC remarkably changed the microstructure of AGLC bioinks. The surface micromorphology changes of the bioinks could cause intense cell responses that influence cell activities, such as adhesion, migrations, and proliferation. The hydrophobic nature of CELC and hydrophilicity (wettability) of the AGLC bioinks decreased as the concentration of CELC increased. The water contact angle increased about 26.13% compared to the AG10:50 bioink. However, all the AGLC bioinks showed hydrophilic characteristics as the contact angle values were less than 90°. The in vitro cell culture study has shown that the prepared bioinks with a lower concentration of the CELC were biocompatible and can support the proliferation of the HeLa cell line, as demonstrated by the MTT assay. Overall, the contributions of this work are represented by (i) developing biomimetic bioinks of AGLC for micro extrusion-based 3D printing, which can be printed and undergo physical crosslinking with Ca^2+^ ions, resulting in well-defined and more stable structures post-printing, and (ii) the characterization of the bioinks and its respective polymers, which showed the wettability, microstructure, thermal stability, and chemical properties.

The careful analyses of all the data obtained from this research indicate that the AGLC01, AGLC05, and AGLC10 bioinks are the most promising bioinks since they exhibited appropriate and suitable thermal stability, wettability, non-cytotoxicity, and surface morphology. They may also create the best environment for cell adhesion and proliferation, among other AGLC bioinks. Considering all the data obtained, we can conclude that the developed AGLC bioinks in this study are promising candidates for applications in tissue engineering. Several suggestions and recommendations are to be considered for future work. The degradation and swollen properties of the AGLC bioinks were not investigated in this work. These properties could influence the mechanical strength and structural integrity and how they change with time. The cytotoxicity of AGLC bioinks and HeLa cell proliferation were carried out in vitro. Moreover, further studies on Hela cell differentiation and behaviour in 3D structures are necessary to prove the promising future of the proposed multi-material bioinks, which are considered important for current research. The biocompatibility of the AGLC hybrid bioinks needs to be evaluated by encapsulating variant types of cells, which is also considered of importance for the current research. Furthermore, the effect of CELC on the viscoelasticity or mechanical properties of the AGLC bioinks should be studied, within which the cells are able to sense and respond to changes in their mechanical environment. Finally, XRD tests could be run to study the semi-crystalline arrangement of the calcium alginate–LC, which is related to the printability. Further study can be conducted to investigate the CELC concentrations of 5–20% *v*/*v* with 10% *w*/*v* of alginate and 50% *w*/*v* of gelatin that might lead to more significant results.

## Figures and Tables

**Table 1:** The proposed concentrations of AG gels.

No	Gel Name	AG (*w*/*v*:*w*/*v*%)
1	AG10:10	10%A:10%G
2	AG10:20	10%A:20%G
3	AG10:30	10%A:30%G
4	AG10:40	10%A:40%G
5	AG10:50	10%A:50%G

**Table 2:** The proposed concentrations of AG10:50-CELC to form AGLC bioinks.

No	Bioink Name	AG-CELC
1	AGLC01	AG10:50-1%
2	AGLC05	AG10:50-5%
3	AGLC10	AG10:50-10%
4	AGLC20	AG10:50-20%
5	AGLC40	AG10:50-40%

**Table 3:** Thermal analysis of the bioinks.

Sample	*T_m_* (°C)	Δ*H* (°C)
AG1050	126.9, 140	29.6
AGLC01	126.9	37.8
AGLC05	121.2	39.4
AGLC10	131, 137	44.3
AGLC20	142.8	49.1
AGLC40	111	42.7

**Figure A1:**
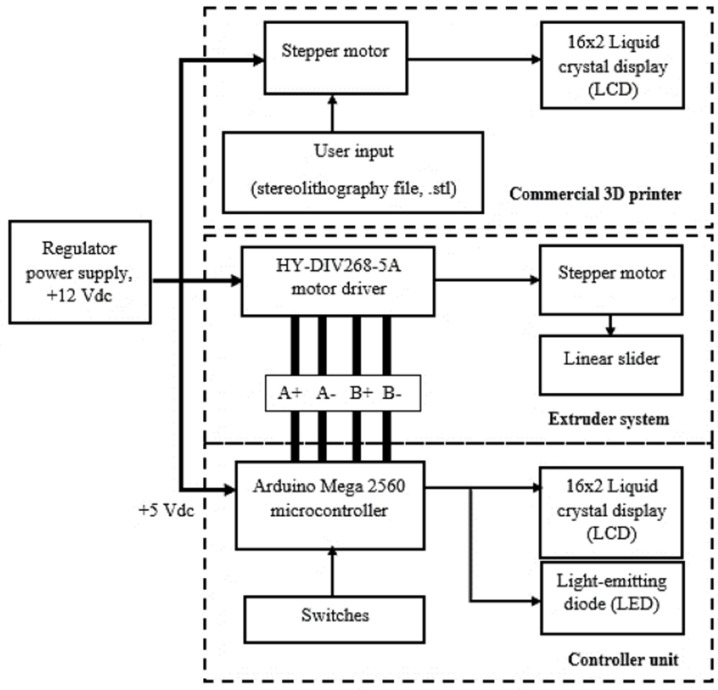
The block diagram of the 3D micro-extrusion bioprinting system design.

## Data Availability

The data presented in this study are available on request from the corresponding author.

## Appendix A

The details about the 3D microextrusion bioprinting system are shown in Appendix A (Algorithm A1 and Figure A1). The pump controller governs the extrusion settings that allow adjusting the extrusion rates values from 1 to 5 mL/min under the diffusion and infusion modes. The extruder machine consists of an extruder motor that allows the mechanical dispensing of the syringe loaded bioink through a nozzle onto a printing platform. The 3D printer moves in the X, Y, and Z coordinate system to create a plane, and is programmed by G-code, a numerical control programming language, to generate specific printed patterns of a square shape. The G-code directs the printing head to create the square shape using a series of G, X, Y, Z commands. The G-code file was transferred to a secure digital (SD) card and inserted into the 3D printer. The designed square shape with dimensions of 3.5 × 3.5 cm was printed on one layer at a printing speed of 30 mm/s. All printing experiments for AG and AGLC bioinks were conducted in a sterilized environment on the clean bench at room temperature (25 °C) using a petri dish as the printing surface. All measurements were performed in triplicate. The parameters setting of the 3D microextrusion-based bioprinting encompasses flow rate of 1–5 mL/min, printing speed of 30 mm/s, and nozzle inner diameter of 1 mm.

**Algorithm A1:** The designed algorithm for the 3D printed shape	
00	**begin**
01	**Header**
02	Setup basic information of the 3D printer including: program name, programmer, date, bioprinter name;
03	**Header-end**
04	**Tooling**
05	Set default tool Set a Programming unit in milimeters;
06	Set a Programming unit in milimeters;
07	Set a path id;
08	**Tooling-end**
09	**Start cutting path id**
10	Perform rapid movement to the nozzle according to X, Y, Z geometric shape;
11	Perform linear interpolation to the nozzle according to X, Y, Z geometric shape;
12	**end cutting path id**
13	**Footer**
14	Turns the spindle off;
15	Move the nozzle to the initial location;
16	Ends the program;
17	**Footer-end**
18	**end**.
